# Supplementary material for: An HSV-2 nucleoside-modified mRNA genital herpes vaccine containing glycoproteins gC, gD, and gE protects mice against HSV-1 genital lesions and latent infection
Source: PLoS Pathog. 2020 Jul 27;16(7):e1008795. doi: 10.1371/journal.ppat.1008795 (PMC7410331; doi:10.1371/journal.ppat.1008795)
Supplement: Attachment — Submitted filename: Response to reviewers comments ver 7.7.2020.docx [file ppat.1008795.s001.docx]

Dear Editor,

Thank you for the opportunity to respond to the reviewers’ comments concerning Pathogens-D-20-01093 “An HSV-2 nucleoside-modified mRNA genital herpes vaccine containing glycoproteins C, D and E protects mice against HSV-1 genital lesions and latent infection”. Below we address each comment and identify locations in the revised manuscript that were modified based on the reviewers’ suggestions. In addition, we made some minor edits throughout the document for clarity. We thank the reviewers for their helpful comments. The revised manuscript is improved because of their input.

**Response to reviewer’s comments**

**Reviewer 1**

Comment: The authors found the RNA vaccination approach to be even better when compared with protein vaccination. The reasons for this are not completely clear. Could it be that the RNA backbone induces a stronger immune response via a TLR activation of antigen-presenting cells? The authors should look at this in more detail.

Response: We added the following statements (lines 310-315) in the revised document: “The induction of T follicular helper cells occurs independent of Toll-like receptors and type 1 interferon (ref 29). The T follicular helper cells stimulate germinal center B cell responses that likely account for the high titers of neutralizing antibodies and antibodies that block C3b binding and IgG Fc binding that are the proposed mechanisms for the improved protection with nucleoside-modified mRNA-LNPs compared to adjuvanted protein.”

Comment: In addition, and very interestingly, the authors report that this HSV-2 specific vaccination strategy also generates cross-reactive antibodies neutralizing HSV-1. It would be interesting to analyze if these neutralizing antibodies protect HSV-1/2 infected animals also in a therapeutic setting, not only in a prophylactic vaccination strategy.

Response: We agree, but these experiments are beyond the scope of this manuscript and will require extensive study. To recognize the importance of this comment, we added the following sentences to the Discussion lines 366-368 in the revised manuscript: “Our efforts with mRNA to date have focused on preventing genital herpes; however, we are also interested in evaluating whether mRNA immunization will be efficacious if used as a therapeutic vaccine (ref 26).”

Comment: Line 260: Fig 4F should be Fig 4C.

Response: Thanks. Change made.

**Reviewer 2**

Comment: The paper is short and it seems there would more opportunity to study mechanisms than is provided.

Response: In response to comments by reviewer 1, we added a mechanism statement (lines 310-315). In response to the comment by reviewer 2, we also added a summary of immune responses comparing the mRNA and protein vaccine based on our prior publication (lines 305-310): “Multiple immune responses were superior in the trivalent mRNA compared with the trivalent protein group, including serum and vaginal IgG ELISA titers, HSV-2 neutralizing antibody titers, antibodies that block gC2 and gE2 immune evasion domains, antibody responses to gD2 epitopes involved in virus entry and cell-to-cell spread, CD4^+^ T follicular helper and germinal center B cell responses. A combination of these immune responses likely accounts for the enhanced performance of the trivalent mRNA vaccine (ref 26).”

Comment: In Figure 2D how were the foci counted? Were multiple sections taken at different parts of the vagina? Was manual or computer aided counting per section done? Was there any variability throughout the vagina? Was there any T cell or other immune cell infiltrate in the vaccine treated mice?

Response: Regarding multiple sections in the vagina and variability throughout the vagina: We added the following statements to Methods in the revised manuscript (lines 434-439): “The vaginal tissues were embedded to allow the entire length of the vagina to be viewed in a single tissue block. Serial 4 µm sections were cut at two depths 100 µm apart. Slides were stained with hematoxylin and eosin and imaged on a Nikon Eclipse 1000 microscope. Images were obtained with 10, 20, and 40X objectives using a Spot model 4.2 CCD color camera and Spot version 4.04 software.

Regarding how were foci counted: We added the following statement to Methods in the revised manuscript (lines 452-453): “To measure the number of HSV-1 antigen foci, slides were imaged with a 2X objective, imported into Image J and manually counted.” We also added the following to Results in the revised manuscript (lines 178-179): “Foci of infection were counted along the entire length of the vagina at two depths separated by 100 μm.”

Regarding did we evaluate for T cell or other immune infiltrates: We performed pan-leukocyte staining with CD45, pan-T cell staining with CD3, and staining for CD4 and CD8 T cells. We do not think the studies are complete at this time. Our plan is to complement these observations using flow cytometry to quantify the number of CD45, CD3, CD4, and CD8 T cells in vaginal tissues, and to evaluate HSV antigen specific CD4 and CD8 T cells in the tissues. We estimate that at least 12 months will be required to complete these studies. Therefore, we propose that these studies are beyond the scope of the current manuscript.

Comment: In Fig 3C: vaginal HSV1 titers at day 2 are on average >1 log higher than in Fig 1E despite using the same inoculum size. Is there a reason for this variability?

Response: We have no explanation for the higher titers in Fig 3C other than the expected variability from one experiment to another. We added the following statement to the revised manuscript (lines 227-229): “HSV-1 day two titers were higher in this experiment than in Fig 1E; however, in both experiments, infection with 2x10^6^ PFU produced higher day two titers for HSV-1 than HSV-2 (Fig 3C).”

Comment: Figure 4B: Are they surprised there is no inoculum dose effect on vaginal viral titers?

Response: Peak vaginal titers in mice generally develop by day two post-infection, which is the day reported in Fig 4B. Our interpretation of the lack of dose-response is that peak titers were similar at the inoculum doses evaluated.

Comment: Are they surprised that the vaginal HSV1 replication out to day 4 in Figure 1E does not result in DRG infection? Do they have an explanation? Presumably this is all local vaginal replication without any axonal-DRG circuit?

Response: We added statements to the Discussion in the revised manuscript (lines 330-334): “The absence of HSV-2 DNA in DRG suggests that vaccine-induced immunity may prevent DRG infection despite local virus replication in genital tract tissues, or possibly that the qPCR assay may not be sufficiently sensitive to detect low level DRG infection despite being able to detect one copy of HSV-2 DNA per 10^5^ copies of adipsin DNA.”

Comment: Given the results in Figures 3C and 4C why not use gD2 alone as an immunogen in an extra arm to see if it mimics the Herpevac results regarding protection against HSV1 versus HSV2?

Response: In the original version of the manuscript and in the revised manuscript, we reference two other studies that reported somewhat better protection against HSV-1 than HSV-2 using gD2 with MPL/alum. We previously reported that the trivalent mRNA vaccine is better at protecting mice against genital HSV-2 infection than gD2 mRNA alone (Awasthi 2019). We are not planning to evaluate gD2 mRNA alone as a vaccine candidate at this time.

Comment: There is no evaluation of T cell responses, even follicular T helper cell responses, in blood or tissue. This might be expected, given the use of adjuvants.

Response: We address this comment in our responses to prior comments by reviewers 1 and 2. We used the same vaccines in the current paper as in our Science Immunology paper where we reported T follicular helper cell responses and HSV-2 antigen-specific CD4 and CD8 T cell responses (ref 26).

Comment: Does PolyC induce interferon?

Response: We added a comment to the revised manuscript (lines 385-387): “Poly(C) RNA was used as a control and is similar to antigen-encoding mRNA in that it does not induce type 1 interferons or proinflammatory cytokines and is immunologically silent (ref 29).”
